# Humeral trochlear morphology does not influence coronoid fractures in elbow dislocation

**DOI:** 10.1186/s40634-023-00571-6

**Published:** 2023-03-15

**Authors:** Paolo Arrigoni, Martina Archinà, Francesco Luceri, Mattia Radici, Carlo Zaolino, Gianluca Folco, Chiara Foschini, Simona Regazzoni, Paul Muriithi Miano, Andrea Zagarella, Alessandra Colozza, Pietro Randelli

**Affiliations:** 1 U.O.C. Clinica Ortopedica e Traumatologica Universitaria CTO, Azienda Socio-Sanitaria Territoriale Centro Specialistico Ortopedico Traumatologico Gaetano Pini-CTO, Via Bignami 1, 20126 Milan, Italy; 2 Residency Program, Università Degli Studi di Milano, Via Mangiagalli 31, 20133 Milan, Italy; 3 S.S. Radiodiagnostica CTO, Azienda Socio-Sanitaria Territoriale Centro Specialistico Ortopedico Traumatologico Gaetano Pini-CTO, Via Bignami 1, 20126 Milan, Italy; 4 Department of Orthopaedic and Trauma Surgery, PCEA Kikuyu Hospital, Nairobi, Kenya; 5 Unità Operativa Ortopedia e Traumatologia, Ospedale Civile di Faenza, Faenza, Italy; 6 Laboratory of Applied Biomechanics, Department of Biomedical Sciences for Health, Università Degli Studi di Milano, Via Mangiagalli 31, 20133 Milan, Italy; 7 Research Center for Adult and Pediatric Rheumatic Diseases (RECAP-RD), Department of Biomedical Sciences for Health, Università Degli Studi di Milano, Via Mangiagalli 31, 20133 Milan, Italy; 8 U.O.C. 1° Clinica Ortopedica, Azienda Socio-Sanitaria Territoriale Centro Specialistico Ortopedico Traumatologico Gaetano Pini-CTO, Piazza Cardinal Ferrari 1, 20122 Milan, Italy

**Keywords:** Simple elbow dislocation, Complex elbow dislocation, Isolated coronoid fracture, Trochlear morphology, Trochlear coverage, Elbow instability, Elbow CT scan, PLRI

## Abstract

**Purpose:**

Traumatic elbow dislocation is the second most frequent joint dislocation, even though the elbow is a congruent and stable joint. Individual variability in anatomical congruence of the elbow and how it relates to simple or complex instability has rarely been studied in the literature; we hypothesized that a greater articular coverage by the humeral trochlea would be more likely to result in complex dislocation. The aim of this study is to analyze trochlear morphology in simple (SED) and complex elbow dislocation (CED), to assess whether the degree of humeroulnar joint congruence influences the incidence of coronoid fractures in elbow dislocation. The secondary goal is to evaluate the association between trochlear morphology and coronoid fracture pattern.

**Methods:**

All the elbow CT scans of the hospital server were retrospectively analyzed. 62 patients were enrolled and so divided in 2 groups: SED and CED with isolated coronoid fracture. Patients who were skeletally immature, presented with other concomitant elbow fractures, or who previously underwent elbow surgery were excluded. The CT scans were performed after closed reduction and prior to further treatment. Coronoid fracture pattern was classified on CT scan according to Regan-Morrey and O’Driscoll classifications; “grade 0” was assigned to SED. Trochlear coverage was measured and expressed as three angles (anterior, posterior, and distal) and their width/depth ratios. Measurements were taken by four different readers and the assessment was repeated after 15 days.

**Results:**

No statistically significant difference was found between humeral trochlear morphology of SED and CED patients. There was no association between morphometric measurements and coronoid fracture pattern. The results are strengthened by a good intra- and inter-reader reproducibility of the CT analysis protocol.

**Conclusions:**

Our study is the first to evaluate the impact of trochlear morphology on elbow instability. Considering the results, other variables may have a greater impact on coronoid bone damage, such as trauma energy or ligamentous hyperlaxity: in particular, we believe that the capsuloligamentous structures of the elbow might contribute in a preponderant way to articular stability. The CT analysis protocol gave excellent results: reproducible, accurate and easy to perform.

**Level of evidence:**

III.

**Supplementary Information:**

The online version contains supplementary material available at 10.1186/s40634-023-00571-6.

## Introduction

Elbow dislocation is a common elbow injury which can be associated with lesions of nearby structures [1]. Dislocation can either be simple, i.e. with no associated fractures, or complex, with one or more concomitant fractures. The majority of movements involving the upper limb, both during daily activities and sports, require a stable, functional, and painless elbow joint. Even though it is one of the most congruent joints, elbow traumatic dislocation is the second most frequent large joint dislocation after shoulder dislocation [2].

Elbow stability is provided by static and dynamic constraints [3]: bony articular surfaces and soft tissues both play a role in determining joint stability. Static stability is guaranteed by bone congruence and the ligamentous complex of the elbow, whereas dynamic stabilizers include muscles, which produce compressive forces. The coronoid process of the ulna constitutes an anterior buttress against varus and valgus stress, as well as posterior translation of the ulna [4]; the anterior band of the medial collateral ligament (A-MCL) and the brachialis muscle insert on the coronoid process [5].

Coronoid fractures can be considered both the direct consequence of posterolateral elbow dislocations and the cause of residual post-traumatic elbow instability; it is commonly accepted that the severity of coronoid fractures correlates directly with the severity of elbow instability. Very few authors have studied isolated coronoid fracture in association to elbow dislocation [6, 7].

While the role of the ulnar greater sigmoid notch congruence has already been studied [8, 9], the relationship between individual anatomical joint congruence and simple (SED) or complex (CED) elbow dislocation remains unclear. The role of distal humeral morphology in humeroulnar instability has not been extensively analyzed.

The aim of the study is to analyze humeral trochlear morphology in patients with SED and CED, under the hypothesis that a greater articular coverage by the trochlea might lead to a greater incidence of CED. The secondary goal is to evaluate the association between trochlear morphology and coronoid fracture pattern in elbow dislocation.

## Materials and methods

In this radiological retrospective study, the primary and secondary goal were assessed through the collaboration between orthopedic surgeons and musculoskeletal radiologists.

An orthopedic surgeon has analyzed all the elbow CT scans of the hospital server. Patients who presented an acute posterolateral elbow dislocation between January 2017 and May 2021 were enrolled. The CT scans were performed after closed reduction and prior to further treatment.

We included 62 skeletally mature patients with either SED or CED with isolated coronoid fractures. Exclusion criteria were skeletally immature patients, fracture of the sublime tubercle with posteromedial instability, transolecranon fracture-dislocation, previous elbow surgery.

Coronoid fractures were classified according to Regan-Morrey [10] and O’Driscoll [11] classifications. SED, namely pure dislocation without fractures, was assigned a “grade 0”. According to O’Driscoll classification, tip fracture was considered as “grade 1”; anteromedial fracture as “grade 2” and basal fracture as “grade 3”. The study group was divided into two subgroups: (1) patients with SED and (2) patients with CED with isolated coronoid fracture.

All the patients gave oral and written consent before enrollment in the study; the University Hospital Centre Review Board approved the study protocol (No. 139/2021).

### Radiological evaluation

**Fig. 1:**
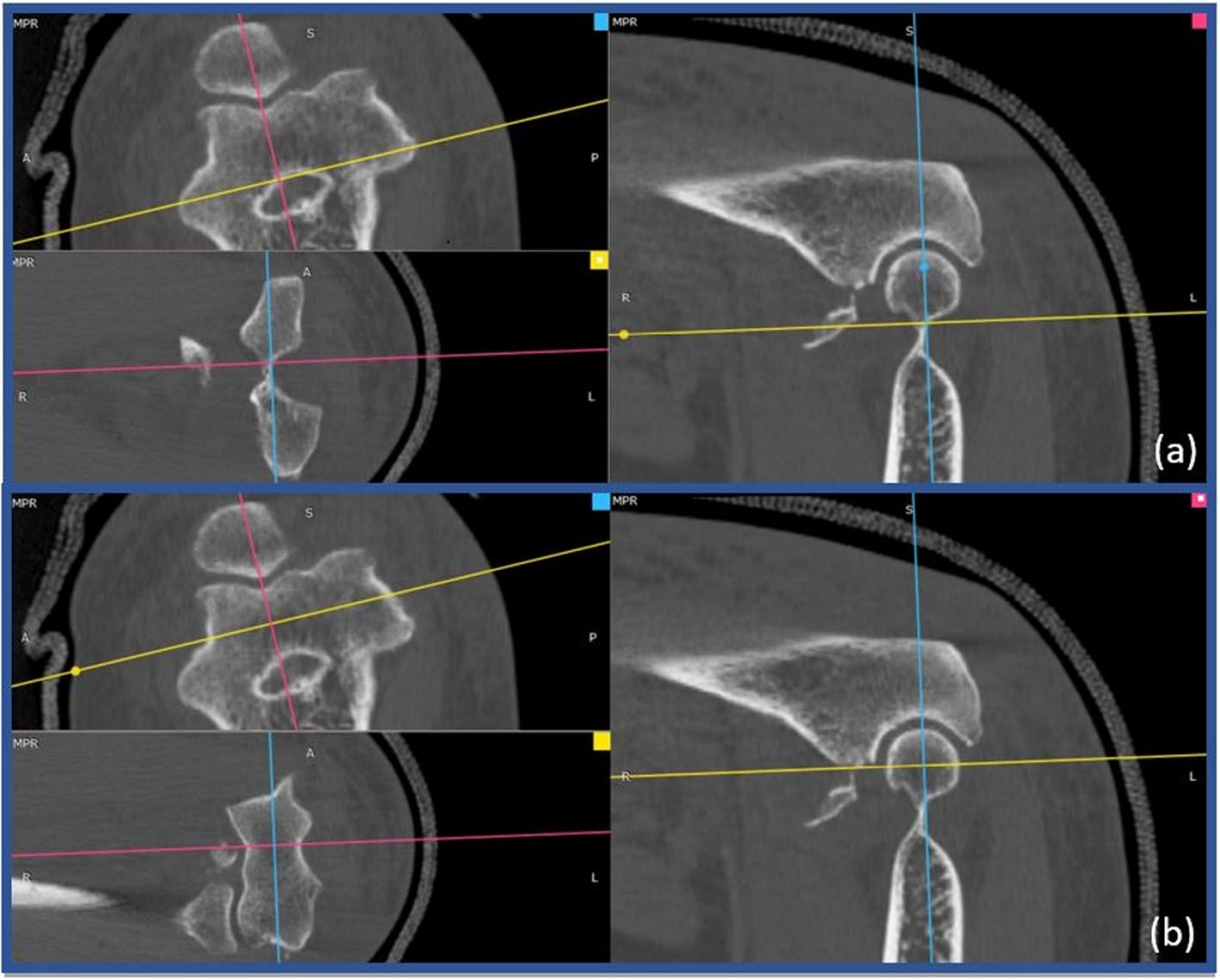
CT Standardization - 3D MPR. (**a**) Position the blue line parallel to humeral diaphysis in sagittal view and parallel to the transepicondylar axis in axial view. Locate the yellow line parallel to the trochlear axis in coronal view. Place the pink line passing through the deepest point of the trochlea in axial and coronal view. **(b)** Position the intersection between the blue line and the yellow one in the center of the humeral trochlea in sagittal view

The Standard CT plane was identified by one of the examiners through RadiAnt DICOM Viewer (Copyright© 2009–2021 Medixant), using three-dimensional multiplanar reconstructions (3D MPR). For the three standard CT planes see Fig. 1.

**Fig. 2:**
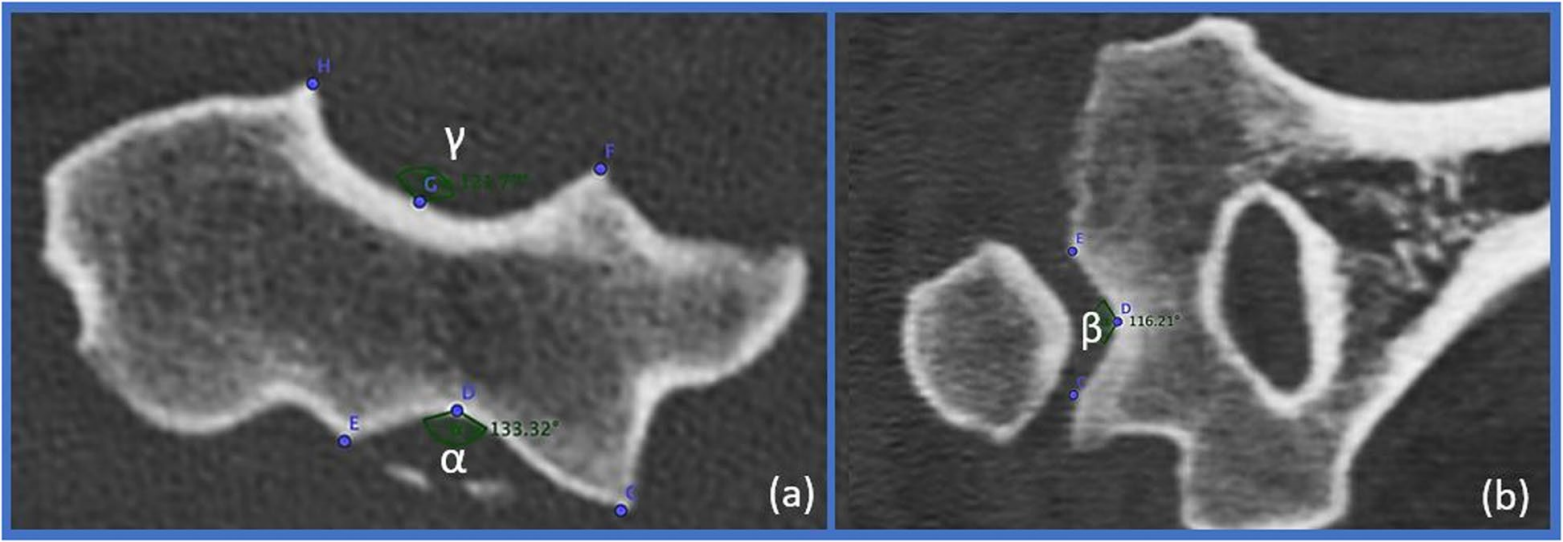
Trochlear angles. (**a**) The anterior α and the posterior γ trochlear angles – axial view. **(b)** The distal β angle – coronal view

**Fig. 3:**
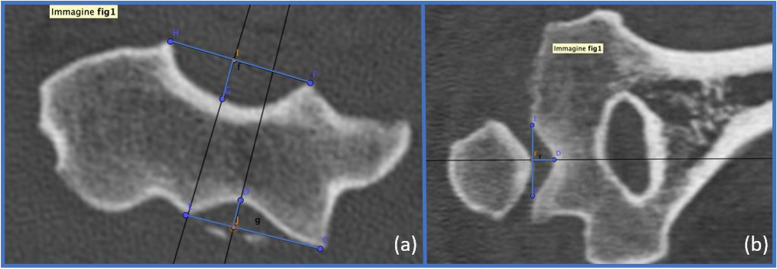
Trochlear ratios. (**a**) The anterior α and the posterior γ width/depth ratios – axial view. **(b)** The distal β width/depth ratio – coronal view

The CT slices were saved as single images (JPEG format) for each patient and later evaluated by four examiners. Trochlear morphology was measured with three dedicated angles: α (anterior trochlear angle, Fig. 2a); β (distal trochlear angle, Fig. 2b) and γ (posterior trochlear angle, Fig. 2a). Their width/depth ratios were also considered (Fig. 3).

CT analysis was performed by four independent examiners, two orthopedic surgeons and two musculoskeletal radiologists. All measurements were repeated after 15 days; the mean of the values was used for the analysis.

**Fig. 4:**
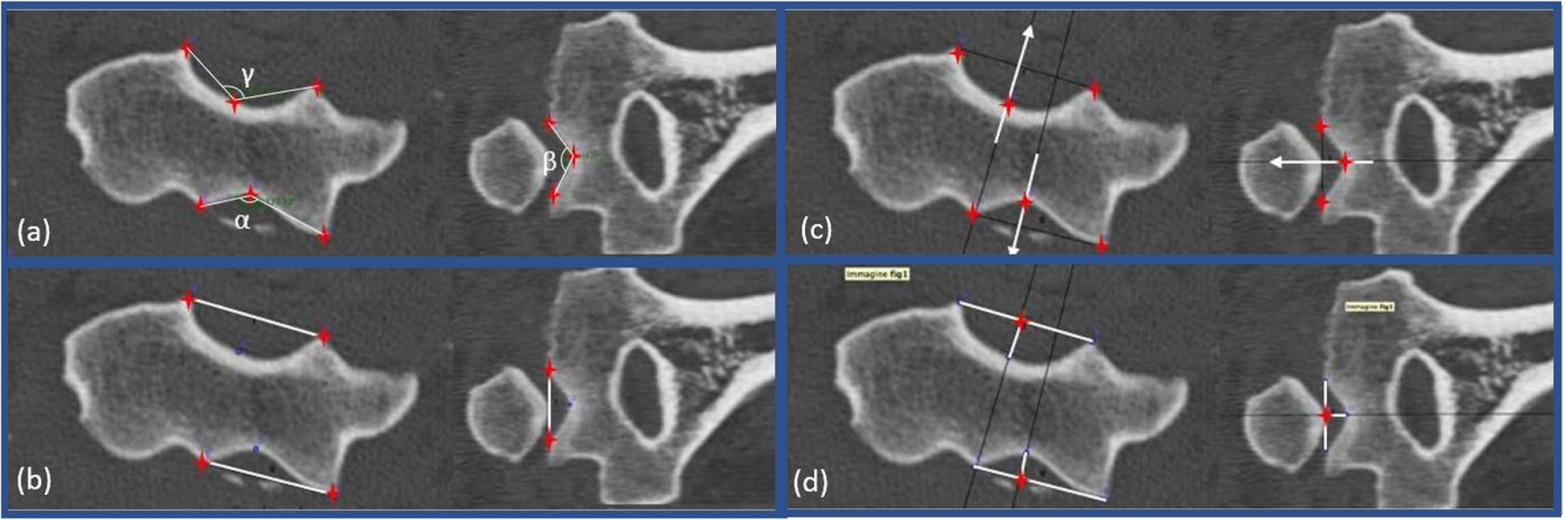
Trochlear groove measurements. (**a**) Both on axial and coronal JPEGs, identify the deepest, the most lateral and most medial points of the trochlear articular surface. Measure anterior α; posterior γ and distal β angles. **(b)** Draw the segment connecting the most lateral and medial point. **(c)** Draw its perpendicular through the deepest point and mark the crossing point of these two lines. **(d)** Calculate the ratio between width and depth of the articular surface in the anterior, posterior, and distal portion of the trochlea in both axial and coronal view

GeoGebra Classic 5.0.639.0-d (Copyright© International GeoGebra Institute) was used to perform all measurements of the humeral trochlear groove. On the JPEG images, first the α and γ angles in axial view were measured. The same was done for the β angle, in coronal view. The ratio between width and depth of the articular surface was calculated in the anterior, posterior, and distal portion of the trochlea in both axial and coronal view (Fig. 4).

Complete radiological analysis can be seen in the Additional file 1.

### Statistical analysis

Statistical analysis was performed using R Statistical Software (version 4.0.0; R Foundation for Statistical Computing) and GraphPad Prism v6.0 software (GraphPad Software Inc.) by a statistician. Continuous variables were expressed as the mean ± standard deviation (SD), as appropriate.

Differences between different group measurements were analyzed with an unpaired Wilcoxon matched-pairs signed-rank test.

The associations between each variable were evaluated with the Pearson or Spearman test, according to the characteristics of the data distribution evaluated using the Shapiro-Wilk normality test. For all analyses, the significance level was set at *p*-value lower than 0.05.

Mangold Reliability Calculator was used to calculate Intraclass Correlation Coefficient (ICC). The inter-observer reproducibility of the CT measurements was evaluated with intra-class correlation coefficient (ICC), which were derived from one-way random-effect analysis of variance. Intra-observer ICC estimates were calculated based on the single measurements, using an absolute-agreement, two-way mixed effects model. Inter-observer ICC estimates were calculated based on the mean value between the two measurements of each observer. The ICC was considered moderate if between 0.500 and 0.749, good if between 0.750 and 0.899, and excellent if above 0.90 according to the Koo and Li scale [12].

## Results

A total of 62 patients were included in the study, with a mean age of 53.18 years (range 17–86). Female to male ratio was 1:1 (31 women and 31 men). 37 (59,68%) presented with CED, while 25 (40,32%) presented with SED.

Radiological measurements are reported in Table 1.

**Table 1:** Variable values distribution. Data reported as mean ± SD, unless indicated otherwise

**Regan-Morrey**	**α**	**β**	**γ**	**α width/depth**	**β width/depth**	**γ width/depth**
**0**	143.0 ± 7.8	137.3 ± 5.8	122.7 ± 7.8	6.5 ± 1.7	5.5 ± 0.9	3.9 ± 0.7
**1**	139.0 ± 7.8	130.2 ± 11.0	121.8 ± 4.3	5.7 ± 1.0	4.9 ± 1.0	3.8 ± 0.4
**2**	142.5 ± 8.4	138.2 ± 8.1	124.9 ± 6.4	6.3 ± 1.5	5.8 ± 1.4	4.1 ± 0.5
**3**	143.4 ± 6.9	136.2 ± 8.8	124.6 ± 8.8	6.6 ± 1.2	5.4 ± 1.2	4.1 ± 0.7
**O’Driscoll**	**α**	**β**	**γ**	**α width/depth**	**β width/depth**	**γ width/depth**
**0**	143.0 ± 7.8	137.3 ± 5.8	122.7 ± 7.8	6.5 ± 1.7	5.5 ± 0.9	3.9 ± 0.7
**1**	138.2 ± 6.9	131.3 ± 11.7	122.2 ± 5.6	5.5 ± 0.9	5.0 ± 1.1	3.9 ± 0.5
**2**	143.1 ± 8.7	137.3 ± 8.1	124.6 ± 5.8	6.4 ± 1.5	5.7 ± 1.4	4.1 ± 0.5
**3**	143.4 ± 6.9	136.2 ± 8.8	124.6 ± 8.8	6.6 ± 1.2	5.4 ± 1.2	4.1 ± 0.7

**Fig. 5:**
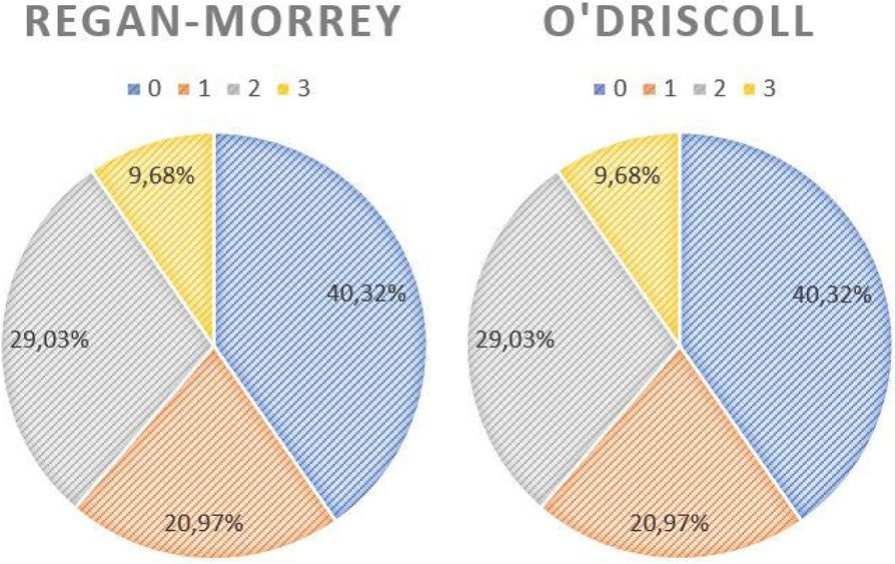
Fracture pattern distribution according to Regan-Morrey and O’Driscoll classifications

Within the CED group, the most frequent fracture pattern was type II fracture (18 cases, 48,65%) according to Regan-Morrey, and anteromedial fracture (18 cases, 48,65%) according to O’Driscoll (Fig. 5).

No statistically significant difference was found between humeral trochlear morphology and the elbow dislocation type (Table 2). There was no association between morphometric measurements and coronoid fracture pattern (Table 3).

**Table 2:** Trochlear morphology in SED and CED groups. Data reported as mean ± SD, unless indicated otherwise

	SED	CED	***p-value***
**Number of patients**	25	37	
**α**	142.6 ± 7.9	141.4 ± 7.7	0.5733
**β**	137.3 ± 4.8	135.0 ± 9.4	0.2732
**γ**	122.6 ± 7.7	122.1 ± 6.5	0.768
**α width/depth**	6.5 ± 1.7	6.1 ± 1.2	0.29
**β width/depth**	5.5 ± 0.8	5.4 ± 1.2	0.6364
**γ width/depth**	3.9 ± 0.6	3.8 ± 0.5	0.8147

**Table 3:** Association between fracture pattern and trochlear morphology

Regan-Morrey	Pearson r	***P*** value	O’Driscoll	Pearson r	P value
**α**	0.08344	0.5191	**α**	0.1055	0.4146
**γ**	0.09856	0.446	**γ**	0.1095	0.3968
**α width/depth**	0.04338	0.7378	**α width/depth**	0.07089	0.584
**β width/depth**	0.1048	0.4174	**β width/depth**	0.07231	0.5765
**γ width/depth**	0.1942	0.1305	**γ width/depth**	0.2075	0.1057
**Regan-Morrey**	**Spearman rho**	**P value**	**O’Driscoll**	**Spearman rho**	**P value**
**β**	−0.002296	0.9859	**β**	−0.02945	0.8203

Intra-observer reproducibility ICCs were between 0.75 and 0.9 (good reproducibility) according to the Koo and Li scale [12], except for the width/depth ratio of the γ angle, which amounted to 0.566 (moderate reproducibility). Inter-observer reproducibility ICCs were between 0.75 and 0.9 (good reproducibility) according to the Koo and Li scale [12], except for the width/depth ratio of the β angle, which amounted to 0.705 (moderate reproducibility). All ICCs values are reported in Table 4.

**Table 4:** Intra- observer and inter-observer reproducibility

variables	ICC intra-observer	ICC inter-observer
**α**	0.89	0.881
**β**	0.883	0.797
**γ**	0.866	0.884
**α width/depth**	0.868	0.847
**β width/depth**	0.845	0.705
**γ width/depth**	0.566	0.814

## Discussion

The main finding of our study is that no statistically significant difference in trochlear morphology has been reported between the SED and CED groups. No statistically significant association between trochlear coverage and coronoid fracture pattern was found. The results are strengthened by a good intra- and inter-reader agreement rate, according to the Koo and Li scale [12]. The most frequent fracture patterns were Regan-Morrey’s type II [10] and O’Driscoll’s anteromedial type [11].

Very few authors have studied CED with isolated coronoid fractures. Kumar et al. [7] have described a series of 6 young patients who got injured by falling on an outstretched hand and were treated by operative fixation. Their study highlighted the fact that only 10% of elbow dislocations presents with isolated coronoid fracture. Foruria et al. [6] included in their study 8 patients with CED with isolated coronoid fractures, out of 28 total patients. In our study we present the largest published series of this kind of injury: out of 62 elbow CT scans performed after closed reduction, 37 presented with CED with isolated coronoid fractures.

This is the first study to investigate elbow instability by analyzing the distal humeral morphology. Similarly, Dejour et al. [13] analyzed femoral trochlear morphology in association with symptomatic patellar instability. In their study, 110 patients with symptomatic patellar instability were enrolled, for a total of 143 knees (33 bilateral cases). The author highlighted that knees with patellar dislocation show morphologic anomalies; trochlear dysplasia, defined by quantitative and qualitative reproducible features, was the main anomaly, present in 85% of cases. As trochlear depth and coverage in elbow fracture-dislocations were analyzed in 62 CT scans, our study confirms that, due to the complexity of the elbow joint, humeroulnar congruence is just one of the factors that determine elbow stability [14]. The main difference with Dejour et al. is that they investigated dislocations secondary to patellar instability, rather than correlating them with a specific fracture pattern. On the other hand, our study strongly hints at the fact that elbow stability is granted by the close interaction of osteoarticular components and soft tissue [14].

Humeral trochlear morphology was previously analyzed in veterinary series [15, 16], through radiographs and CT scans. These authors never defined specific angles to study trochlear congruence. Our CT analysis protocol was newly designed to evaluate quantity and quality of trochlear coverage through the α, β, and γ angles. These three angles represent the global congruence of the trochlea. The 3D MPR, performed through RadiAnt DICOM Viewer, allowed to properly standardize the axial, coronal, and sagittal projections for each DICOM file.

A more congruent trochlea should contribute to provide more resistance to dislocation in the setting of a posterolateral rotatory instability (PLRI) mechanism [17]. Because elbow subluxations rarely represent an indication to perform a CT scan, it is difficult to study whether these patients would present with a more congruent trochlea, preventing complete dislocation.

CT scan is a fundamental tool in evaluation of elbow instability. This series is also the first to evaluate CT scans both on the humeral and ulnar side. Our data confirm the important role of the mix between articular congruence of the humeroulnar joint and soft tissues towards elbow stability, ruling out an isolated contribution by humeral bony anatomy. Lesions and ligamentous laxity of the lateral collateral ligament (LCL) complex can influence the fracture pattern in case of elbow dislocation. Joint hyperlaxity predisposes individuals to musculoskeletal lesions [18]. Age and sex are probably the most important factors affecting non-genetic joint hypermobility [19, 20].

It is widely accepted that trauma energy influences the fracture pattern in case of injury [21]. A fall on the outstretched hand is a common event as cause of acute elbow dislocations. Approximately 40% of these injuries occur during sports; gymnastics, wrestling, basketball, and football [22]. Multiple elbow fractures, often comminuted, are associated with high-energy injuries. CED with isolated coronoid fracture usually occurs in medium- to low-energy trauma. The impact of trauma energy in CED with isolated coronoid fracture has not been studied yet.

There are some limitations to our study; it is a retrospective series and lacks the clinical evaluation of patients. However, it still remains the largest studied group of CED with isolated coronoid fracture patients, observed from an innovative point of view – humeral morphology – and through a reliable and valid CT analysis protocol.

This study opens the door to further investigations. As ligamentous hyperlaxity can promote joint instability, this should be investigated. Assessment of ligament integrity and radiological signs of instability could also be evaluated through an MRI analysis of the elbow, rather than via CT scan.

## Conclusions

The novel CT parameters proposed in our study are simple, reliable, and reproducible. These features make them a promising tool for CT evaluation of elbow instability.

No differences in humeral trochlear anatomy were reported between simple and complex elbow dislocations. The study hypothesis is rejected, as a more congruent trochlea is not associated to a greater likelihood of CED.

## Supplementary Information

**Additional file 1.** Radiological evaluation.
