# Supplementary material for: Humeral trochlear morphology does not influence coronoid fractures in elbow dislocation
Source: J Exp Orthop. 2023 Mar 15;10:25. doi: 10.1186/s40634-023-00571-6 (PMC10014637; doi:10.1186/s40634-023-00571-6)
Supplement: Supplementary file 1 — Additional file 1. Radiological evaluation. [file 40634_2023_571_MOESM1_ESM.docx]

*Additional file 1 Radiological evaluation*

The Standard CT plane was identified by one of the readers through RadiAnt DICOM Viewer (Copyright © 2009 – 2021 Medixant), using three-dimensional multiplanar reconstructions (3D MPR). Three lines were identified (Figure 1a):

• Blue line: parallel to the humeral diaphysis, passing through the center of the trochlea in sagittal view, and parallel to the trans-epicondylar axis, passing through the medial and the lateral epicondyle in axial view.

• Yellow line: parallel to the trochlear axis, passing through the center of the trochlea in coronal view.

• Pink line: parallel to the humeral axis in coronal view, passing through the deepest point of the trochlea in axial and coronal view.

The intersection between the blue and the yellow line was then positioned through the center of the humeral trochlea in a sagittal view (Figure 1b). The CT slices were saved as single images (JPEG format) for each patient and later evaluated by four readers.

Trochlear coverage was measured with three dedicated angles:

• α (anterior trochlear angle): angle defined by three points, the deepest and the most lateral and most medial points of the anterior trochlea in axial view (Figure 2a).

• β (distal trochlear angle): angle defined by three points, the deepest and the most lateral and most medial points of the articular surface of the inferior trochlea in coronal view (Figure 2b).

• γ (posterior trochlear angle): angle defined by three points, the deepest and the most lateral and most medial points of the articular surface of the posterior trochlea in axial view (Figure 2a).

Their width/depth ratios were also considered:

• α or anterior width/depth ratio: ratio between the segment that connects the most lateral and the most medial points of the articular surface of the anterior trochlea (width) and the segment perpendicular to the width, passing through the deepest point of the articular surface (Figure 3a).

• β or distal width/depth ratio: ratio between the segment that connects the most lateral and the most medial points of the articular surface of the inferior trochlea (width) and the segment perpendicular to the width, passing through the deepest point of the articular surface (Figure 3b).

• γ or posterior width/depth ratio: ratio between the segment that connects the most lateral and the most medial points of the articular surface of the posterior trochlea (width) and the segment perpendicular to the width, passing through the deepest point of the articular surface (Figure 3a).

CT analysis was performed by four independent examiners, two orthopedic surgeons and two musculoskeletal radiologists. All measurements were repeated after 15 days; the mean of the values was used for the analysis.

GeoGebra Classic 5.0.639.0-d (Copyright © International GeoGebra Institute) was used to perform all measurements of the humeral trochlear groove. On the axial and coronal JPEG images, three reference points were identified: the deepest, the most lateral and the most medial ones of the trochlear articular surface. First, the α and γ angles in axial view were measured. The same was done for the β angle, in coronal view (Figure 4a). A segment connecting the most lateral and the most medial points of the trochlea was drawn (Figure 4b); then, its perpendicular through the deepest point was also traced (Figure 4c). Finally, we marked the crossing point of these two lines. The ratio between width and depth of the articular surface was calculated in the anterior, posterior, and distal portion of the trochlea in both axial and coronal view (Figure 4d).
